# Experimental and Monte Carlo based dosimetric investigation of a novel 3 mm radiosurgery 3 MV beam using the microSilicon detector

**DOI:** 10.1002/acm2.14388

**Published:** 2024-05-19

**Authors:** Katrin Saße, Karina Albers, Peter Douglas Klassen, Neelan J. Marianyagam, Georg Weidlich, M. Bret Schneider, Steven Chang, John Adler, Björn Poppe, Hui Khee Looe, Daniela Eulenstein

**Affiliations:** ^1^ University Clinic for Medical Radiation Physics, Medical Campus Pius Hospital Carl von Ossietzky University Oldenburg Germany; ^2^ Bonifatius Hospital Lingen Lingen Germany; ^3^ Department of Neurosurgery Stanford University School of Medicine Palo Alto California USA; ^4^ ZAP Surgical Systems San Carlos California USA; ^5^ PTW‐Freiburg Freiburg Germany

**Keywords:** microSilicon detector, Monte Carlo simulations, radiosurgery, small field dosimetry, volume effect

## Abstract

**Background:**

The ZAP‐X system is a novel gyroscopic radiosurgical system based on a 3 MV linear accelerator and collimator cones with a diameter between 4 and 25 mm. Advances in imaging modalities to detect small and early‐stage pathologies allow for an early and less invasive treatment, where a smaller collimator matching the anatomical target could provide better sparing of surrounding healthy tissue.

**Purpose:**

A novel 3 mm collimator cone for the ZAP‐X was developed. This study aims to investigate the usability of a commercial diode detector (microSilicon) for the dosimetric characterization of this small collimator cone; and to investigate the underlying small field perturbation effects.

**Methods:**

Profile measurements in five depths as well as PDD and output ratio measurements were performed with a microSilicon detector and radiochromic EBT3 films. In addition, comprehensive Monte Carlo simulations were performed to validate the measurement observations and to quantify the perturbation effects of the microSilicon detector in these extremely small field conditions.

**Results:**

It is shown that the microSilicon detector enables an accurate dosimetric characterization of the 3 mm beam. The profile parameters, such as the FWHM and 20%–80% penumbra width, agree within 0.1 to 0.2 mm between film and detector measurements. The output ratios agree within the measurement uncertainty between microSilicon detector and films, whereas the comparisons of the PDD results show good agreement with the Monte Carlo simulations. The analysis of the perturbation factors of the microSilicon detector reveals a small field correction factor of approximately 3% for the 3 mm circular beam and a correction factor smaller than 1.5% for field diameters above 3 mm.

**Conclusions:**

It could be shown that the microSilicon detector is well‐suitable for the characterization of the new 3 mm circular beam of the ZAP‐X system.

## 1 INTRODUCTION

There is an unmet medical need in stereotactic radiosurgery for smaller collimators. The fundamental tenet of stereotactic radiosurgery (SRS) is to deliver a high radiation dose to the target while minimizing exposure to surrounding healthy tissue. In part, this is achieved by matching the size of the collimator to that of the anatomical target.^1^ So far, targets with small spatial dimensions could be treated with the smallest conventionally available collimator of 4 mm in diameter,^2^ even though some targets were reported to have smaller diameters of only approximately 2.5 mm.^3^

The ability to deliver smaller, more precise radiosurgical treatments allows us to make use of the ever‐improving ability to detect and precisely localize increasingly small pathologies.^4^ Furthermore, for specialized clinical applications, such as non‐destructive “radiomodulation”^5^, ^6^ the beam diameter to the target is essential to achieve the desired therapeutic effect and at the same time to minimize off‐target effects.

Ultrasmall collimators have previously been explored^7^ and implemented on a C‐arm linear accelerator with a 3 mm cone using the historic Wendell Lutz multi‐arc treatment technique. On the radiosurgical system ZAP‐X (Zap Surgical Systems, Inc. of San Carlos, California) currently, irradiations are carried out with circular collimators with diameters between 4 and 25 mm. In this work, the first attempt to further reduce the field diameter to 3 mm has been described. A comprehensive dosimetric characterization has been performed with a diode detector and radiochromic films. Furthermore, Monte Carlo simulations were performed to quantify the underlying perturbation effects associated with the diode detector in this extremely narrow beam. Thereby, the applicability of the detector to commission the newly introduced beam diameter was evaluated and analyzed.

## 2 MATERIALS AND METHODS

### 2.1 ZAP‐X

**FIGURE 1:**
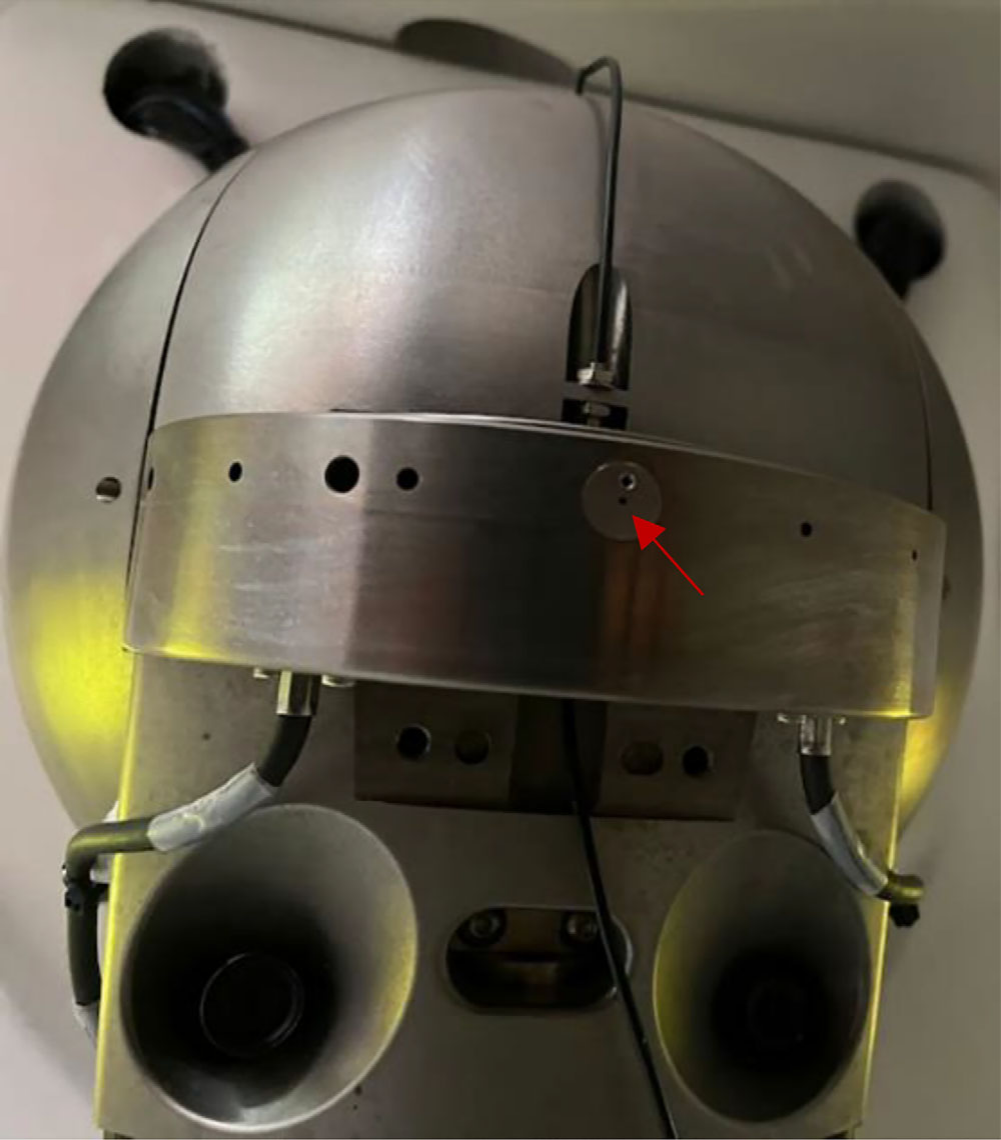
A 3 mm insert placed inside the 25 mm cone in the ZAP‐X collimator wheel as indicated by the arrow.

The study was performed at a ZAP‐X system. The self‐shielding 3 MV linear accelerator (linac) with circular collimators is a dedicated machine for stereotactic radiosurgery of brain lesions. To ensure accurate patient positioning, a three‐dimensional image registration based on planar kilovoltage (kV) images is implemented. A collimator wheel (see Figure 1) with holes cut through produces eight circular beam diameters between 4 and 25 mm.^8^, ^9^

### 2.2 The novel 3 mm cone collimator

To realize the new 3 mm collimator size, an additional tungsten conical insert was fitted into the existing 25 mm cone within the collimator wheel (see Figure 1). Therefore, no major modifications were necessary to introduce the new beam. After measurements were completed, the insert could be easily removed.

### 2.3 Measurements

All measurements were performed in a 3D motorized MP3‐XS water phantom with a TANDEM electrometer (PTW Freiburg, Germany). The water phantom was attached to the couch with a special frame and leveled manually. The phantom and the electrometer were controlled with the BEAMSCAN v4.5. software. The water level in the phantom was verified every 30 min and the water tank was refilled to ensure constant source‐to‐surface distance (SSD) during the entire measurement duration.

In this work, the microSilicon dector (Type 60023, PTW Freiburg, Germany), which is an unshielded silicon diode detector was used. The detector has been shown to be suitable for small‐field dosimetry in previous studies.^10^, ^11^, ^12^, ^13^, ^14^ It operates at 0 V and has an active diameter of 1.5 mm. It was orientated parallel to the beam, using the TRUFIX detector positioning system in the water phantom. All measurement conditions were chosen to comply with the requirements for beam commissioning of the dedicated treatment planning system (TPS). PDD and profile measurements were performed at SSD of 450 mm. Profiles were obtained at five water depths (7, 50, 100, 200, and 250 mm) and two directions, defined as wheel and ortho directions. The wheel plane is directed along the collimator wheel, while the second direction is perpendicular to it. The profile and PDD measurements using the microSilicon detector were acquired with a step size of 0.2 mm and a measuring time of 0.5 s per measuring point.

Output ratio (OR) measurements were performed at SSD of 443 mm in 7 mm water depth. The output ratio was calculated according to Equation 1:

(1) $$OR\;=\frac{M_{det}^{f_{clin}}}{M_{det}^{f_{msr}}}\;$$

where $M_{det}^{f_{clin}}$ is the detector measured signal/MU at the clinical (clin) field sizes 3, 4, 5, and 25 mm. $M_{det}^{f_{msr}}$ is the detector‐measured signal/MU at the machine specific reference (msr) field. For the ZAP‐X system, the largest cone of 25 mm was used as the msr field according to the TRS 483 formalism. For all collimator sizes, the beam inclination was checked before the measurement and the detector was centered according to the radiation field. The largest measured inclination was 0.41° with the 3 mm cone. This small inclination was taken into account by the water phantom software by moving the detector along the measured inclination.

Besides the microSilicon detector, measurements were also performed with EBT3 radiochromic films. Film pieces with sizes of 50 mm × 50 mm were positioned on a thin PMMA plate attached to the TRUFIX system. The 2D beam profiles and the output factors were measured in 7 mm water depth. Each measurement was repeated five times, where each film was irradiated with 200 MU which corresponds to approximately 2 Gy with the 25 mm reference cone at the measurement position. The films were scanned using an Epson 10000 XL scanner with a resolution of 600 dpi at least 24 h after the exposure. All software‐based auto corrections were disabled, and the files were saved as uncompressed tiff images. The film analysis was performed in Matlab using the red channel.^13^, ^15^, ^16^, ^17^

The film calibration was performed with a 6 MV photon beam at an Elekta Synergy linear accelerator for the same film batch number (05122102) using 10 dose values between 0 and 2 Gy. Due to the low energy dependence of the film response,^18^ the difference between the 3 MV and 6 MV beam qualities were assumed to be negligible. The film calibration curve was fitted to a third‐degree polynomial function. The noise in the film measurements was reduced using a median filter across a homogeneously irradiated area of about 80 × 80 pixels (corresponding to an area of 27.1 mm × 27.1 mm) for the calibration films and 5 × 5 (0.42 mm × 0.42 mm) pixels for the output measurements. All measurements were carried out in two separate sessions 3 months apart.

### 2.4 Monte Carlo simulations

To model the radiation beams, individual virtual fluence source ψ(r) for each cone diameter was derived based on the convolution model:

(2) $$D\;\left(r\right)=\;\psi \left(r\right)*K_{D}\left(r\right)$$

where D(r) is the dose distribution in water and $K_{D}\left(r\right)$ is the so‐called dose deposition kernel. For each field size, the D(r) has been measured at 7 mm water depth using EBT3 films as described previously. The energy‐dependent function $K_{D}\left(r\right)$ was simulated using a 0.2 × 0.2 mm^2^ pencil beam for the 3 MV photon spectrum provided by ZAP‐X. For each field size, the ψ(r) was obtained by solving Equation 1 via deconvolution with the corresponding D(r) with $K_{D}\left(r\right)$ using the van‐Cittert iterative algorithm.^19^ The same approach was applied in our previous studies to model narrow split beam geometry^20^ and small fields of conventional linac.^21^

**FIGURE 2:**
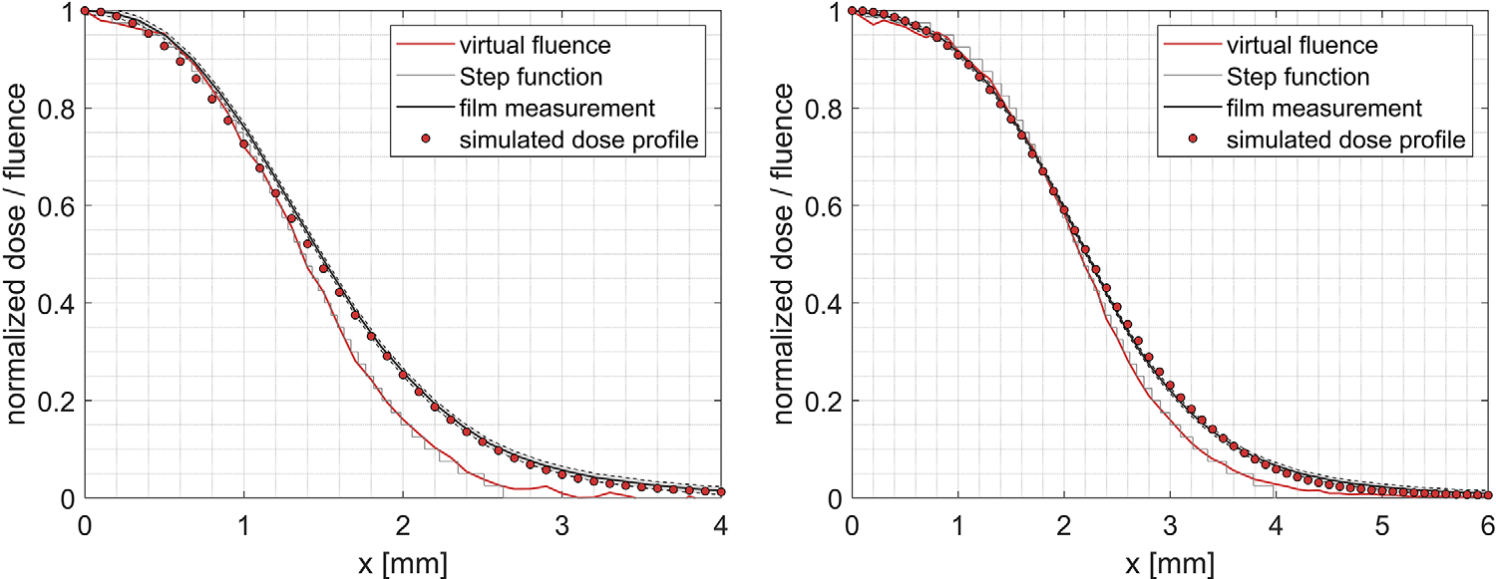
Measured EBT3 dose profiles in 7 mm depth for the 3 mm (left) and 4 mm (right) collimator (black lines). The virtual photon fluence profiles (red lines) were derived according to Equation 1 and implemented as the superposition of individual weighted step functions (gray lines). Using these implemented virtual sources, the corresponding profiles were simulated (red closed circles) and compared to the film measurements (black line, with 1 standard deviation indicated by dashed lines).

Figure 2 shows exemplarily the measured D(r) (black lines) at 7 mm water depth and the derived photon fluence profile ψ(r) (red lines) for the 3 and 4 mm cones. These sources were implemented in the EGSnrc Monte Carlo code using the user code *egs_chamber* as a superposition of weighted step functions (gray lines). Further simulation settings used in EGSnrs are given in Table 1. To validate the implemented virtual fluence sources, the dose profiles at the same measurement depth were simulated (red closed circles) and compared to the original film measurements.

**TABLE 1:** Simulation settings in EGSnrc used in this work.

Item name	Description
Code	EGSnrc/eges_chamber
ECUt	512 keV
PCUT	10 keV
Variance reduction techniques	
Photon‐cross‐section‐enhancement (XCSE)	XCSE factor = 512
Russian Roulette	Rejection factor = 521, E < 512 keV
Intermedia Phase‐space‐scoring (IPSS)	
Number of histories	100 single batches with 1 × 10^10^ histories each

**FIGURE 3:**
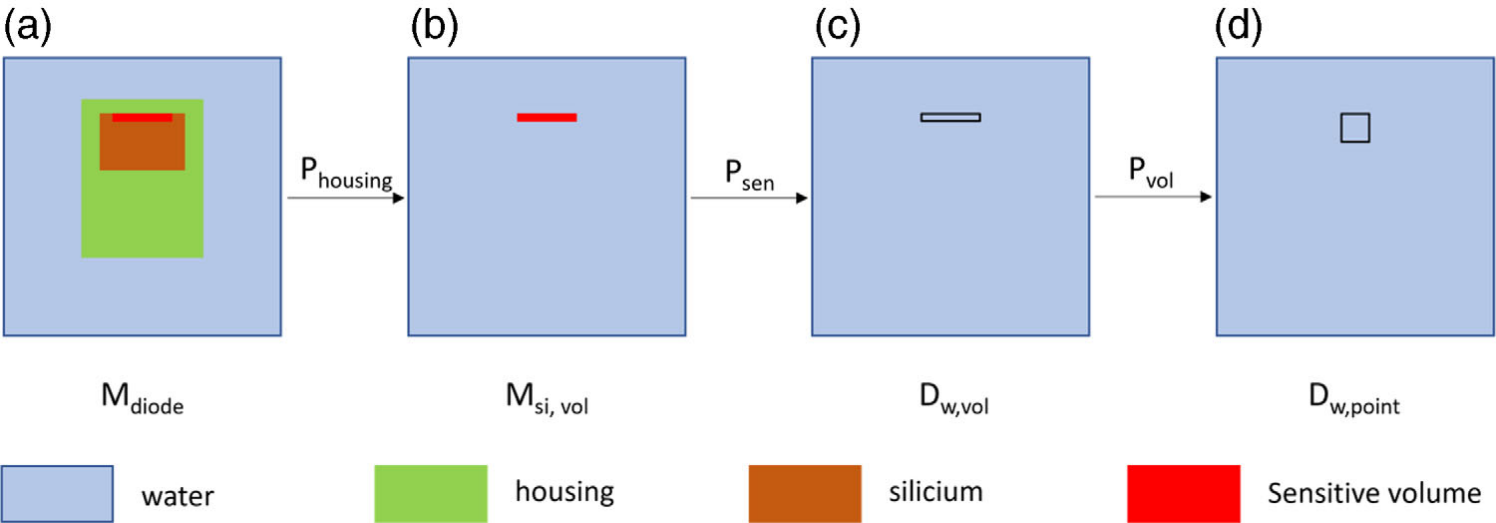
Step‐wise modification of microSilicon detector model in the detailed Monte Carlo simulations to quantify the associated small field perturbation effects (not to scale).

Using these validated virtual fluence sources, the behavior of the microSilicon detector in these small field sizes was modeled and studied. The detector was modelled according to the manufacturer's blueprint. First, the signal profiles M(r) of the microSilicon detector at 7 mm water depth were simulated with a step size of 0.1 mm. Second, the underlying small field perturbation effects associated with the microSilicon detector were individually quantified with detailed Monte Carlo simulations according to the approach presented in Weber et al.^14^ Thereby, four different models were implemented as presented in Figure 3. In the first step (3a) the complete microSilicon detector was modeled. In the second step (3b), the detector housing and including the silicon chip, except the sensitive volume, was replaced by water. In the third step (3c), the material of the sensitive ship was also replaced by water. The last step (3d) represents the absorbed dose‐to‐water at the point of measurement, which in this case was modeled as a 0.02 cm × 0.02 cm 0.02 cm water voxel.

For these simulations, the detector models were always positioned at the center of the beam. The associated perturbation correction factors $P_{housing}^{f_{clin},f_{msr}}$, $P_{sens}^{f_{clin},f_{msr}}$, $P_{vol}^{f_{clin},f_{msr}}$ and the output correction factor were calculated according to Equations 3a–3c.

(3a) $$P_{housing}^{f_{clin},f_{msr}}=\frac{M_{si,vol\;}^{f_{clin}}/M_{diode\;}^{f_{clin}}\;}{M_{si,vol\;}^{f_{msr}}/M_{diode\;}^{f_{msr}}\;}\;$$

(3b) $$P_{sens}^{f_{clin},f_{msr}}=\;\frac{D_{w,vol\;}^{f_{clin}}/M_{si,vol\;}^{f_{clin}}\;}{D_{w,vol\;}^{f_{msr}}/M_{si,\;vol\;}^{f_{msr}}\;}$$

(3c) $$P_{vol}^{f_{clin},f_{msr}}=\frac{D_{w,point\;}^{f_{clin}}/D_{w,vol\;}^{f_{clin}}\;}{D_{w,point\;}^{f_{msr}}/D_{w,\;vol\;}^{f_{msr}}\;}\;$$

where, the *M* values refer to the simulated detector signal in terms of absorbed dose in the detector sensitive volume and the *D* values refer to the simulated absorbed dose‐to‐water in the water voxel (point, step 3d) or the sensitive volume replaced by water (vol, step 3c). The perturbation factors associated with each small clinical (clin) field size were calculated as the ratios between the subsequent steps, normalized to that at the msr field.

## 3 RESULTS

### 3.1 Profiles

**FIGURE 4:**
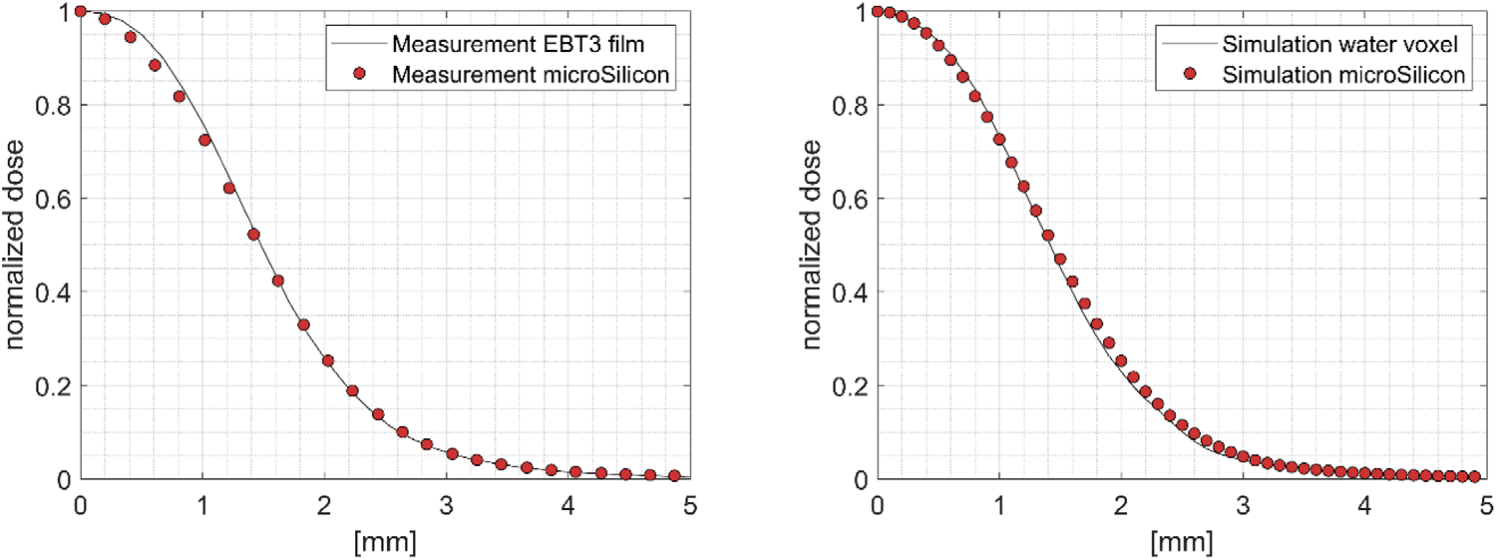
Left: Comparison of the measured profiles of the new 3 mm collimator size at 7 mm water depth with the microSilicon detector and EBT3 films. Right: Simulated dose‐to‐water and the signal profile of the microSilicon detector for the same conditions.

The measured profile using the microSilicon detector along the ortho axis at 7 mm water depth is compared to the film measurement in Figure 4 (left panel) for the new 3 mm diameter beam.

The evaluated dosimetric field size at this depth, defined as full‐width‐at‐half‐maximum (FWHM) values, resulted in 3.0 and 2.9 mm from the microSilicon measurements in the ortho and wheel directions, respectively, as tabulated in Table 1. The dosimetric field size measured with the films amounts to 3.0 mm. Furthermore, the penumbra widths measured using the microSilicon detector, defined as 20%−80% distance, are 1.4 and 1.3 mm in the ortho and wheel directions, respectively. The penumbra width obtained with films amounts to 1.3 mm. Thereby, an agreement between microSilicon measurement and EBT3 can be observed. The same agreement was observed in the Monte Carlo simulations by comparing the simulated dose profiles and the detector measurements (Figure 4, right panel).

The comparison of the measured field sizes and penumbra widths for the 3 mm collimator using the microSilicon detector and films at five different depths is tabulated in Table 2. Besides, the comparisons between the microSilicon measurements acquired at two independent sessions (I and II) are also presented demonstrating the reproducibility of the collimator and measurement setups.

**TABLE 2:** Dosimetric field sizes and penumbra widths derived from the profile measurements obtained with the microSilicon detector and EBT3 films for the 3 mm collimator size.

		Field size [mm]			Penumbra left [mm]		Penumbra right [mm]		
Water depth [mm]	Axis	Session I micro Silicon	Session II micro Silicon	Film	Session I micro Silicon	Session II micro Silicon	Session I micro Silicon	Session II micro Silicon	Penumbra film [mm]
7	Ortho	3.0	3.0	3.0	1.4	1.4	1.4	1.3	1.3
	Wheel	2.9	2.9		1.3	1.3	1.4	1.4	
50	Ortho	3.3	3.3	3.3	1.6	1.6	1.5	1.5	1.5
	Wheel	3.3	3.3		1.5	1.5	1.5	1.5	
100	Ortho	3.7	3.7	3.6	1.8	1.8	1.7	1.7	1.7
	Wheel	3.6	3.6		1.7	1.7	1.7	1.7	
200	Ortho	4.4	4.5	4.4	2.2	2.2	2.2	2.1	2.1
	Wheel	4.4	4.4		2.1	2.1	2.2	2.2	
250	Ortho	4.9	4.8	4.8	2.4	2.4	2.3	2.3	2.3
	Wheel	4.7	4.8		2.3	2.3	2.4	2.4	

### 3.2 PDD

**FIGURE 5:**
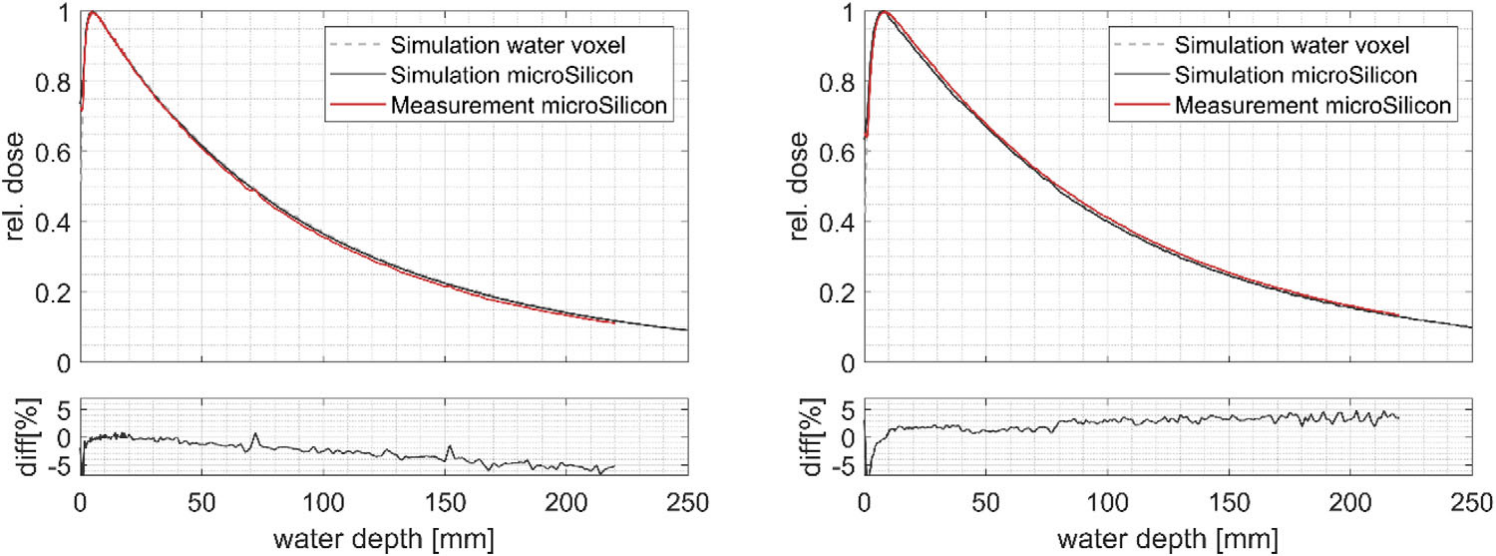
PDD measured with the microSilicon detector and simulated with water voxel and the microSilicon detector model. The difference in the lower panels indicates the deviation between the microSilicon measurement and Monte Carlo simulation with the detector model. Left: 3 mm collimator size, right: 25 mm collimator size.

Figure 5 shows the measured PDD using the microSilicon detector for the 3 and 25 mm collimator sizes. Due to the reduction of phantom scattering at the 3 mm collimator sizes, the depth of dose maximum is shifted toward a lower depth of 4.8 mm as compared to 7.8 mm at the reference 25 mm collimator diameter. The dose fall‐off at the 3 mm field size is also steeper than that of the 25 mm reference collimator size. At 100 mm depth, the percentage dose for the 3 mm collimator size was measured to be 35.6%, as compared to 41.2% for the 25 mm collimator size. This difference is smaller when comparing between 3 and 4 mm collimator sizes, where the percentage dose measured at 100 mm depth for the 4 mm collimator size corresponds to 36.7%. The difference between the Monte Carlo simulations and measurement is less than 5% and 7% for the 25 and 3 mm collimator diameters, respectively, where the largest deviation was observed at the build‐up region and large depths (beyond 150 mm). It is noteworthy that the virtual photon sources in the Monte Carlo simulations do not account for contamination electrons that partly contributed to the discrepancy between measurements and simulations in the build‐up region. Furthermore, the agreement between the simulated and measured PDD of the microSilicon detector indicates the suitability of the detector to measure the PDD correctly even in this very small field size.

### 3.3 Output factors

**FIGURE 6:**
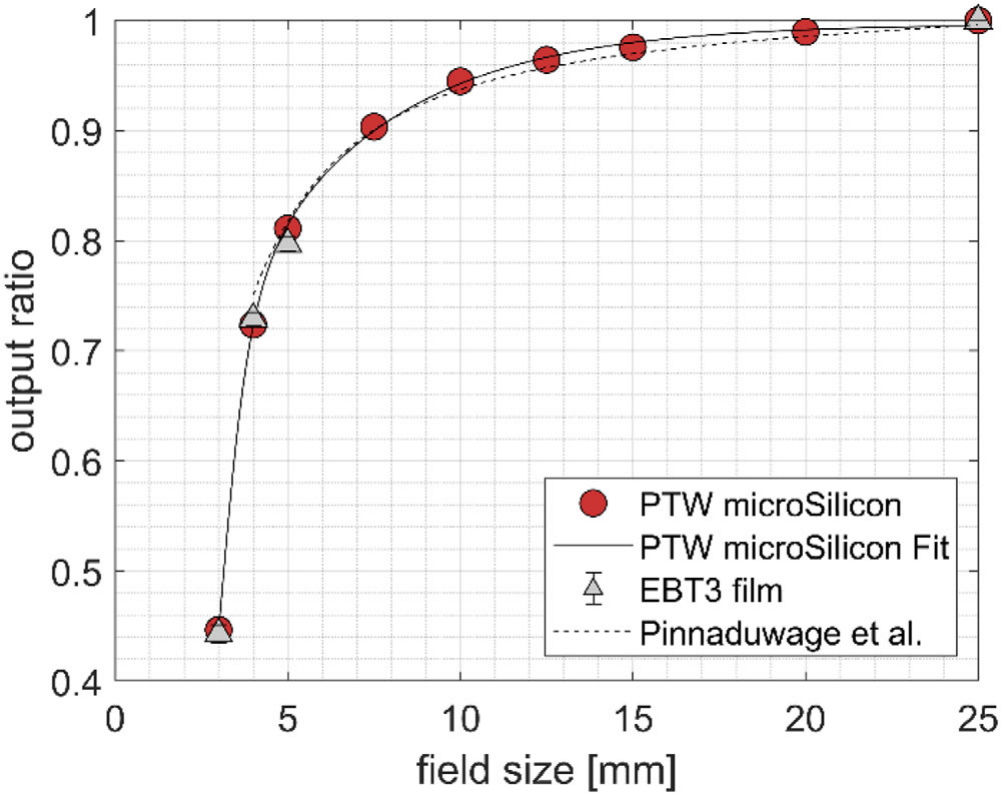
Measured OR with the microSilicon detector and the output factors measured with the EBT3 films obtained in this study. The microSilicon detector data down to 4 mm collimator size has been fitted according to Equation 4 to ease the comparison with the data of Pinnaduwage et al.

The uncorrected detector OR (Equation 4) obtained with the microSilicon detector as well as the output factors obtained with EBT3 films are shown in Figure 6. For comparison, the fit function of the output factors reported by Pinnaduwage according to Equation 4 is also shown:

(4) $$\mathrm{OF}\;\left(s\right)=\;\frac{p\cdot s^{n}}{a^{n}+s^{n}}+s\cdot \left(1-\exp\left(-b\cdot s\right)\right)$$

where *s* is defined as the nominal field size and *a*, *b*, *p*, *s*, and *n* are the fit parameters. The measured OR of the microSilicon detector from this study has been also fitted with this fit function as presented in the plot. The agreement between the two fit functions in the nominal field size range from 4 to 25 mm is better than 0.03 showing a good consistency with the results of Pinnaduwage et al.^22^

The measured OR using the microSilicon detector are 0.447 ± 0.004, 0.724 ± 0.003, and 0.811 ± 0.003 for the collimator sizes of 3, 4, and 5 mm, respectively; while the output factors obtained with films resulted in 0.443 ± 0.008, 0.729 ± 0.006, and 0.797 ± 0.007 for the collimator sizes of 3, 4, and 5 mm, respectively.

### 3.4 Small field perturbation effects of the microSilicon detector

**FIGURE 7:**
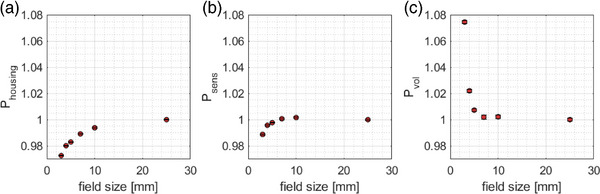
Simulated small perturbation correction factors of the microSilicon detector calculated according to Equation 2.

The simulated small field perturbation correction factors for the microSilicon detector are shown in Figure 7. On the one hand, with decreasing field size, the volume‐averaging effect increases as the field dimension approaches the diameter of the sensitive volume (1.5 mm), which results in a detector under‐response. P_vol_ starts to deviate from unity at cone diameters smaller than 10 mm, where at 4 mm diameter, P_vol_ amounts to 1.022 (Figure 7c). At the 3 mm collimator diameter, a sharp increase of P_vol_ to 1.075 could be observed. On the other hand, the housing of the detector causes an over‐response, that can be attributed to the enhanced density of the housing material, including the epoxy casting resin with a mass density of 1.15 g/cm. At 4 mm and 3 mm collimator diameters, the P_housing_ amounts to 0.980 and 0.973, respectively. Last, the material of the sensitive volume (silicon with a mass density of 1.4 g/cm) also results in a detector over‐response, where for the 3 mm cone, the associated correction factor lies at 0.989.

**FIGURE 8:**
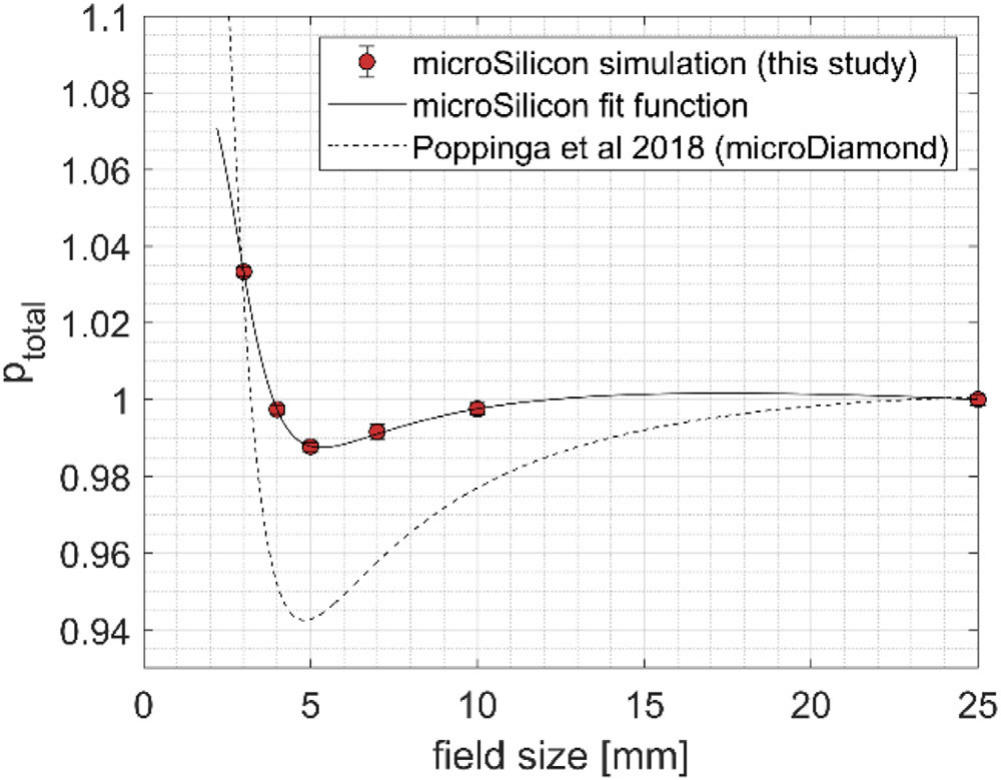
Total perturbation correction factors for the microSilicon for cone sizes between 3 and 25 mm. For comparison, the correction factors for the microDiamond detector as reported in Poppinga et al.^15^ with a turning point also at around 5 mm are presented.

The field size‐dependent total correction factors between 3 and 25 mm cone diameter are presented in Figure 8. Due to the competing effect of the density perturbation (causing an over‐response) and the volume‐averaging effect (causing an under‐response), the required total correction factor amounts to 0.988, 0.998, and 1.033 for the collimator sizes of 5, 4, and 3 mm, respectively, with a turning point at 5 mm collimator size. Similar behavior has been reported previously^15^ for the microDiamond detector (PTW Freiburg, Germany) as shown in the same figure for comparison.

## 4 DISCUSSION

In this study, the dosimetric characteristics of the new 3 mm cone for the ZAP‐X system have been studied comprehensively. Thereby, the lateral profiles, PDD, and output ratios were acquired using the microSilicon detector. For comparison, the lateral profiles and output factor also was obtained with EBT3 films. Complementary Monte Carlo simulations were performed, on the one hand, to validate the measurement results and observations, and on the other hand, to provide insights on the perturbation effects associated with the microSilicon detector for measurements in these very small field sizes.

The dosimetric field size of the 3 mm cone at 7 mm depth measured with EBT3 films was found to be 3.0 mm in both the ortho and wheel directions, whereas the values of 2.9 and 3.0 mm were determined with the microSilicon detector for the ortho and wheel directions, respectively. The penumbra width at 7 mm depth measured with the microSilicon detector amounts to 1.35 ± 0.05 mm, which agrees with film measurements (1.3 mm).

For the 3 mm cone collimator, the measured maximum depth of the PDD is shifted to a lower depth of 4.8 mm, as compared to 8.6 mm at the reference 25 mm field diameter. A steeper dose fall‐off of the smaller collimator field size can also be observed due to the reduction of phantom scattering. Pinnaduwage et al. reported comparable values for the measured dose for the 25 mm beam diameter. For both the 3 and 25 mm field diameters, agreement within 5% and 7%, respectively, between the simulated and measured microSilicon PDD was obtained. Furthermore, comparisons between the simulated microSilicon PDD and the unperturbed PDD in water demonstrated that the microSilicon can be used for PDD measurements of the reference field size as well as of the new 3 mm collimator diameter without further corrections. This is in agreement with the results reported by Akino et al.,^23^ who demonstrated that the microSilicon detector is suitable for PDD measurements at large depths in small field sizes.

The measured output factor using EBT3 films of the 3 mm cone amounts to 0.443 ± 0.008. For comparison, the output factor at the current smallest clinical field diameter of 4 mm is 0.729 ± 0.006, indicating a reduction of output by 28.5 percentage points between the two smallest field diameters. The OR of the 3‐ and 4‐mm collimator diameters measured with the microSilicon detector amounts to 0.447 ± 0.004 and 0.724 ± 0.003, respectively, which lie within the uncertainty of the measurements.

Altogether, the results in this study indicated that the microSilicon used in this study is a suitable detector for the commissioning of the novel 3 mm radiation beam. These findings might appear to be surprising at first as the physical dimensions (diameter) of the detector's sensitive volume is half of the width of the 3 mm radiation beam itself. To provide more insights into these observations, the perturbation effects associated with the microSilicon detector have been quantified using detailed Monte Carlo simulations. This was accomplished by separating the perturbations attributed to the volume‐averaging effect, the material density of the detector housing; and the material of the sensitive volume itself, by step‐wise modification of the detector model.

Due to the 1.5 mm diameter of the sensitive volume of the microSilicon detector, the volume‐averaging effect causes 7.5% under‐estimation of the output at the 3 mm cone diameter, that is, P_vol_ amounts to 1.075 as shown in Figure 7c. Nevertheless, this amount of perturbation is not apparent both in the profile (Figure 4) as well as the OR measurements (Figure 6), suggesting that this effect is being negated partly by other perturbation effects. Our Monte Carlo simulations demonstrated that an effect opposing the volume‐averaging is mainly caused by the detector's housing. Similar behavior has been reported for the microSilicon detector in small field sizes of conventional C‐arm linear accelerators.^14^ At 3 mm collimator diameter, this density perturbation associated with the detector's housing causes a signal over‐estimation of around 3%, that is P_housing_ amounts to 0.9725. Furthermore, the material of the sensitive volume itself also causes additional over‐response, although the magnitude is lower (around 1% at the 3 mm collimator diameter). These competing effects can be best seen in the computed field size‐dependent total correction factors as presented in Figure 8 with a turning point at 5 mm collimator diameter. With decreasing collimator size from 5 mm, the volume‐averaging effect increases rapidly as shown in Figure 7c. At the new 3 mm collimator size, the volume‐averaging effect becomes more dominant resulting in the output correction factor of 1.034. This study is the first to observe this turning point for the microSilicon detector. So far, this effect has only been reported for the microDiamond detector.^15^ Furthermore, the resulting perturbation dominated by the volume‐averaging effect of the microSilicon detector can be observed in Figure 4 as the microSilicon detector's profiles are slightly broader than the film profiles. It is worth noting that the turning point for the microSilicon detector results in an output correction factor larger than unity for 3 mm field size. All previous studies^10^, ^11^, ^12^, ^13^, ^14^ on the microSilicon detector have analyzed field sizes larger than 4 mm and determined correction factors smaller than unity, which are consistent with our results for larger field diameters.

## 5 CONCLUSION

In this study, a 3 mm collimator for the ZAP‐X system was dosimetrically characterized for the first time. It could be shown, by comparison to EBT3 film measurements, that the microSilicon detector in combination with a motorized water phantom is well suited to acquire commissioning data such as lateral profiles, PDD as well as output factor of the novel 3 mm field diameter. Although the active volume of the microSilicon detector has a diameter of 1.5 mm, Monte Carlo simulations demonstrated that the microSilicon detector requires only 3.3% correction for the new field diameter at the field center, which is in accordance with the 5% limit for correction factors as recommended by the TRS 483.^24^ Detailed Monte Carlo quantification of the individual small field perturbation effects reveals the competing effect between volume‐averaging and density perturbations. This study provided deeper insights into the detector's behavior in these very small fields used for high‐precision radiosurgery.

## AUTHOR CONTRIBUTIONS

Katrin Saße performed all measurements, contributed to the simulations, and wrote the manuscript. Karina Albers contributed to the measurements and performed the Monte Carlo simulations and evaluations of the simulations results. Peter Douglas Klassen contributed to the scientific discussions and review of the manuscript. Neelen J. Marianyagam performed some measurements and contributed to the scientific discussions and review of the manuscript. Georg Weidlich contributed to the development of the 3 mm cone, scientific discussions, and review of the manuscript. M. Bret Schneider contributed to the development of the 3 mm cone and its medical use. Steven Chang contributed to the scientific discussions and review of the manuscript. John Adler contributed to the scientific discussions and review of the manuscript. Björn Poppe contributed to the scientific discussions and review of the manuscript. Hui Khee Looe contributed to the conception of the work, the Monte Cao simulations, and the writing of the manuscript. Daniela Poppinga contributed to the conception of the work, evaluation of the results, and writing the manuscript.

## CONFLICT OF INTEREST STATEMENT

Daniela Eulenstein is employee of PTW Freiburg. Georg Weidlich, M. Bret Schneider, and John Adler are employees of ZAP Surgical Systems.

## References

[1] Friehs GM , Park MC , Goldman MA , Zerris VA , Norén G , Sampath P . Stereotactic radiosurgery for functional disorders. Neurosurgical Focus. 2007;23(6):E2. 10.3171/FOC-07/12/E3 18081480

[2] Kondziolka D , Perez B , Flickinger JC , Habeck M , Lunsford LD . Gamma knife radiosurgery for trigeminal neuralgia: results and expectations. Arch Neurol. 1998;55(12):1524‐1529. 9865796 10.1001/archneur.55.12.1524

[3] Erbay SH , Bhadelia RA , O'Callaghan M , et al. Nerve atrophy in severe trigeminal neuralgia: noninvasive confirmation at MR imaging—initial experience. Radiology. 2006;238(2):689‐692. 16436823 10.1148/radiol.2382042214

[4] Su JH , Thomas FT , Kasoff WS , et al. Thalamus Optimized Multi Atlas Segmentation (THOMAS): fast, fully automated segmentation of thalamic nuclei from structural MRI. Neuroimage. 2019;194:272‐282. 30894331 10.1016/j.neuroimage.2019.03.021 PMC6536348

[5] Schneider MB , Walcott B , Adler Jr JR . Neuromodulation via focal radiation: radiomodulation update. Cureus. 2021;13(4):e14700. 33927960 10.7759/cureus.14700 PMC8076105

[6] Wang X , Chang C , Adler Jr JR , et al. A randomized blinded trial of nucleus accumbens ablation to treat opiate dependence in humans: location correlates with outcome. Cureus. 2012;4(6):e49.

[7] De Salles AA , Melega WP , Laćan G , Steele LJ , Solberg TD . Radiosurgery performed with the aid of a 3‐mm collimator in the subthalamic nucleus and substantia nigra of the vervet monkey. J Neurosurg. 2001;95(6):990‐997. 11765845 10.3171/jns.2001.95.6.0990

[8] Weidlich GA , Schneider MB , Adler Jr JR . Self‐shielding analysis of the Zap‐X system. Cureus. 2017;9(12):e1917. 29441251 10.7759/cureus.1917 PMC5800761

[9] Weidlich GA , Schneider MB , Adler Jr JR . Characterization of a novel revolving radiation collimator. Cureus. 2018;10(2):e2146. 29632755 10.7759/cureus.2146 PMC5880589

[10] Delbaere A , Younes T , Simon L , Khamphan C , Vieillevigne L . Field output correction factors and electron fluence perturbation of the microSilicon and microSilicon X detectors. Phys Med Biol. 2022;67(8):08NT01. 10.1088/1361-6560/ac5e5e 35294937

[11] Francescon P , Kilby W , Noll J , Satariano N , Orlandi C . Small field dosimetry correction factors for circular and MLC shaped fields with the CyberKnife M6 System: evaluation of the PTW 60023 microSilicon detector. Phys Med Biol. 2020;65(1):01NT01. 10.1088/1361-6560/ab6154 31829983

[12] McGrath AN , Golmakani S , Williams TJ . Determination of correction factors in small MLC‐defined fields for the Razor and microSilicon diode detectors and evaluation of the suitability of the IAEA TRS‐483 protocol for multiple detectors. J Appl Clin Medical Phys. 2022;23(7):e13657. 10.1002/acm2.13657 PMC9278669 35652320

[13] Schönfeld AB , Poppinga D , Kranzer R , et al. Characterization of the new microSilicon diode detector. Med Phys. 2019;46(9):4257‐4262. 31309594 10.1002/mp.13710 PMC6852691

[14] Weber C , Kranzer R , Weidner J , et al. Small field output correction factors of the microSilicon detector and a deeper understanding of their origin by quantifying perturbation factors. Med Phys. 2020;47(7):3165‐3173. 32196683 10.1002/mp.14149 PMC7496769

[15] Poppinga D , Delfs B , Meyners J , Harder D , Poppe B , Looe HK . The output factor correction as function of the photon beam field size–direct measurement and calculation from the lateral dose response functions of gas‐filled and solid detectors. Zeitschrift für Medizinische Physik. 2018;28(3):224‐235. 28869164 10.1016/j.zemedi.2017.07.006

[16] Poppinga D , Schoenfeld AA , Doerner K‐J , Blanck O , Harder D , Poppe B . A new correction method serving to eliminate the parabola effect of flatbed scanners used in radiochromic film dosimetry. Med Phys. 2014;41(2):021707. 24506598 10.1118/1.4861098

[17] Schoenfeld AA , Poppinga D , Harder D , Doerner K‐J , Poppe B . The artefacts of radiochromic film dosimetry with flatbed scanners and their causation by light scattering from radiation‐induced polymers. Phys Med Biol. 2014;59(13):3575. 24909235 10.1088/0031-9155/59/13/3575

[18] Massillon‐JL G , Chiu‐Tsao S‐T , Domingo‐Munoz I , Chan MF . Energy dependence of the new Gafchromic EBT3 film: dose response curves for 50 kV, 6 and 15 MV X‐ray beams. Int J Med Phys Clin Eng Radiat Oncol. 2012;1(2):60‐65. 10.1118/1.4735153 PMC13387173 28517140

[19] Looe HK , Harder D , Poppe B . Understanding the lateral dose response functions of high‐resolution photon detectors by reverse Monte Carlo and deconvolution analysis. Phys Med Biol. 2015;60(16):6585. 26267311 10.1088/0031-9155/60/16/6585

[20] Delfs B , Blum I , Tekin T , et al. The role of the construction and sensitive volume of compact ionization chambers on the magnetic field‐dependent dose response. Med Phys. 2021;48(8):4572‐4585. 34032298 10.1002/mp.14994

[21] Blum I , Tekin T , Delfs B , et al. The dose response of PTW microDiamond and microSilicon in transverse magnetic field under small field conditions. Phys Med Biol. 2021;66(15):155003. 10.1088/1361-6560/ac0f2e 34181591

[22] Pinnaduwage DS , Srivastava SP , Yan X , et al. Small‐field beam data acquisition, detector dependency, and film‐based validation for a novel self‐shielded stereotactic radiosurgery system. Med Phys. 2021;48(10):6121‐6136. 34260069 10.1002/mp.15091

[23] Akino Y , Fujiwara M , Okamura K , et al. Characterization of a microSilicon diode detector for small‐field photon beam dosimetry. J Radiat Res (Tokyo). 2020;61(3):410‐418. 32211851 10.1093/jrr/rraa010 PMC7299273

[24] Palmans H , Andreo P , Huq MS , Seuntjens J , Christaki KE , Meghzifene A . Dosimetry of smalll static fields used in external photon beam radiotherapy: summary of TRS‐483, the IAEA‐AAPM international Code of Practice for reference and relative dose determination. Med Phys. 2017;45(11):e1123‐e1145. 10.1002/mp.13208 30247757

